# Electrochemical, surface, and theoretical investigations of palm kernel shell extract as an effective inhibitor for carbon-steel corrosion in 1 M HCl solution

**DOI:** 10.1007/s11356-024-35159-9

**Published:** 2024-10-08

**Authors:** Omotayo Sanni, Jianwei Ren, Tien-Chien Jen

**Affiliations:** 1 Department of Mechanical Engineering Science, University of Johannesburg, Cnr Kingsway and University Roads, Johannesburg, 2092 South Africa; 2 Department of Chemical Engineering, Faculty of Engineering, Built Environment and Information Technology, University of Pretoria, Pretoria, 0002 South Africa

**Keywords:** Green corrosion inhibitors, Palm kernel shell extract, Carbon steel, Acidic corrosion

## Abstract

Herein, we employed palm kernel shell extract (PKSE) as an eco-friendly inhibitor for carbon steel in acidic-induced corrosion. The corrosion inhibition of PKSE on carbon steel in 1 M HCI solution was investigated by electrochemical impedance spectroscopy, weight loss, and potentiodynamic polarization measurements. The surface was characterized by scanning electron microscopy equipped with energy-dispersive X-ray spectroscopy. Moreover, the elastic modulus and hardness tests were conducted. Weight loss measurements revealed that the optimum concentration of inhibitors is 500 ppm with 95.3% inhibition efficiency in 1 M HCl solution. Electrochemical results showed that the inhibitor could exhibit excellent corrosion inhibition performance and displayed mixed-type inhibition. The electrochemical impedance spectroscopy analysis shows that the inhibition performance increases by increasing the concentration of PKSE*.* The surface studies ensure the PKSE effectiveness in carbon steel surface damage reduction. Also, the adsorption of PKSE molecules on the carbon steel surface occurs according to the Langmuir isotherm model. The primary goal of this investigation was the utilization of palm kernel shell extract as corrosion inhibitor for 1018 low carbon steel in 1 M HCl solution, which highlights its novelty. The present results will be helpful to uncover the versatile importance of palm kernel shell compounds in the corrosion inhibition process.

## Introduction

Carbon steel is widely used in buildings, bridges, boilers, oil and gas pipeline manufacturing, etc., owing to its low cost and mechanical characteristics (Rbaa et al. 2020; Yang 2021). The carbon steel should be pretreated with acid to remove its oxidation films before manufacturing, referred to as acid descaling or pickling. Owing to the acid fierceness, besides oxidation films, carbon steel ineluctably tends to corrode, particularly, in chemical and petrochemical industries (Toghan et al. 2021; Xu et al. 2023a; Bidi et al. 2020; Alamiery et al. 2021); carbon steel is in direct contact with strong acidic solution, resulting in the reduction of their service life and causing serious accidents. The media used to study the corrosion inhibition ability in different literature were mainly 1.0 M HCl solution, primarily because most metal chlorides are easily soluble in water. The treatment of rusty metal with HCl solution is capable of dissolving a wide range of corrosion products on metal.

In contrast, the HCl solution is used for chemical cleaning processes as an acid detergent with a concentration of exactly 1.0 M (Chen et al. 2022). Also, in carbon steel/HCl solution systems, the reaction rate of iron oxide with HCl is faster than that of iron oxide with H_2_SO_4_, which is far more than that of iron oxide with HNO_3_, HClO_4_, citric acid, formic acid, and acetic acid. Therefore, it is essential to note that the corrosion rate of carbon steel in HCl is more than twice that of carbon steel in H_2_SO_4_, and choosing the suitable corrosion inhibitor is crucial during the pickling process. Therefore, developing efficient technologies to inhibit or prevent carbon steel corrosion is crucial. Among other reported techniques in preventing carbon steel from corrosion, corrosion inhibitor is more effective compared to other methods due to its simplicity and “in situ” advantage without disrupting the ongoing process (Tiwari et al. 2021; Hajjaji et al. 2021). The synthetic organic compound has been evaluated as a corrosion inhibitor, and study into their evaluation and synthesis is ongoing. These compounds exhibit high toxicity to human life and the environment, though they exhibit good anticorrosion properties. It was reported that such inhibitors adsorbed on carbon steel occur by sharing lone pair electrons of O, S, or N atoms to the unoccupied orbital’s of the metallic surface. The films formed act as the protective layers that separate the metal from the corrosive solutions. It is reported that nitrogen-containing inhibitors are imidazole and its derivatives (Kerru et al. 2020; Wei et al. 2020; Avdeev and Kuznetsov 2021) and amine and its derivatives (El Ibrahimi et al. 2020; Verma et al. 2020). The sulfur-containing inhibitor contains thiourea and its derivatives (Huong et al. 2020; Zhang et al. 2021; Lavanya et al. 2021). Though these inhibitors exhibit excellent corrosion inhibitive performance, their low biodegradability, high cost, and rigorous synthesis protocols hamper their sustainable application. Severe environmental issues have also caused the scientific and economic communities to focus on utilizing eco-friendly compounds without inherent toxicity as effective corrosion inhibitors.

Moreover, since the whole idea of metal protection is anchored on economic gain and environmental sustainability, the substance to be used as corrosion inhibitor must be cheap, readily available, and environmentally friendly. Hence, research activities are geared toward finding a replacement for inorganic metal corrosion inhibitors. Agricultural waste is one of the sources of cheap, readily available, and nontoxic green corrosion inhibitors. Due to the increasing awareness of sustainable development, biomass waste has been regarded as a desirable material for inhibiting metals against corrosion, which may also be an effective solution for garbage sorting and recycling. Accordingly, agricultural waste contains many active compounds such as carboxylic acids, bioamines, polysaccharides, and polyphenols that could be extracted by simple less expensive procedures. Extracts from wastes have been widely reported as an effective and good metal corrosion inhibitors in various corrosive media such as banana leaves extracts (Guo et al. 2021; Raghavendra et al. 2021; Ananaba and Okonkwo 2023), *Ammi visnaga* extract (Zaher et al. 2022), Cannabis sativa extract (Haldhar et al. 2021), pumpkin leaf extract (Ren et al. 2024); Justicia gendarussa plant extract (Gunasekaran et al. 2009), *Pyracantha fortuneana* alcohol extracts (Tan et al. 2024a); pomelo peel extract (Lin et al. 2021), longan extract (Cao 2022), *Moringa oleifera* leaf extract (Allaoui et al. 2020; Mejeh et al. 2020; Didouh et al. 2023), apple fruit extract (Umoren et al. 2015), carbon dots (Xu et al. 2023b); honeysuckle extract (Sun et al. 2023); wood hibiscus leaf extract (Xu et al. 2023a); barbarum leaf extract (Gu et al. 2023), extract of Pistacia khinjuk (Soltani et al. 2020), almond waste extract (Izuagie et al. 2023; Batah et al. 2022); feverfew waste extract (Zhou et al. 2023); eggshell extract (Sanni et al. 2023); Jordanian agro-waste extract (Hayajneh et al. 2023); walnut extract (Shahmoradi et al. 2021); soybean extract (Wan et al. 2022). Jmiai et al. (2021) investigated jujube shell extract to mitigate copper corrosion in 1 M HCl solution resulting in an efficiency of 91% (2 g/L extract). Li et al. (2020) employed walnut green husk extract to inhibit cold rolled steel in 3.0 M H_3_PO_4_ solution. Although insufficient inhibition efficiency was observed using only husk extract, 97.2% efficiency was attained by the synergistic effect between the extract and sodium lignosulfonate. Kouache et al. (2022) extracted polyphenols from *Inula viscosa* and employed the extract to prevent the corrosion of X70 steel in 1 M HCl solution, yielding a maximum efficiency of 92%.

The research interest has been necessitated by the fact that the present corrosion inhibitors in the market for the protection of carbon steel in the acidic media are hazardous to the environment and thus compromise safety and sustainability drives (Sanni et al. 2023). There is, therefore, the need to develop inhibitors that are eco-friendly and sustainable. There is, therefore, the need to develop inhibitors that are eco-friendly and sustainable. The most efficient agro-waste extracts reported in the literature contain tannins, phenolic acids, and alkaloids. The aromatic structure, mostly rich in nitrogen and oxygen content, adsorbed on the surface of the metal prevents the metal from corroding. The palm kernel shell extract has been used in treating some diseases such as musculoskeletal disorders and restoring bone fractures in traditional medicine. Its anti-inflammatory effect was reported by Mahmud et al. (2020), and its pyrolysis by Su et al. (2022). Products from palm kernel are in constant use in the society owing to increase in demand for palm oil. According to Dit (2007) “The excess quantity of palm kernel shell was about 350,000 tons per year with an assumption that 25% was available for sale, 50% of palm kernel production was used within the palm oil industry and 25% by other industries.” Palm oil industry is one of the largest sources of biomass. Among various wastes from palm oil processing, palm kernel shell is an oil palm biomass with high potential to be applied as a source of energy, given its high caloric value and distinctive physical properties (Handaya et al. 2022). The ultrastructure, physicochemical, thermal characteristics, and applications of PKSE have been extensively studied by different researchers (Okoroigwe and Saffron 2012; Ntenga et al. 2018; Zainal et al. 2024); the results revealed the potential of palm kernel shell as corrosion inhibitor.

Based on the compounds identified, and since this extract has not yet been reported for carbon steel 1018 in one molar hydrochloric acid media as a corrosion inhibitor, palm kernel shell extract has been selected for the present study. The carbon steel 1018 was chosen as the test material owing to its wide utilization as construction material in gas-treating plants and susceptibility to severe corrosion. Hence, in this work, the inhibitory effect of palm kernel shell extract (PKSE) was evaluated using the potentiodynamic polarization, weight-loss, and EIS techniques. The adsorption mechanism of PKSE on the metal surface was further studied by examining the various adsorption isotherms, and the hardness test was assessed. The structure of the PKSE is well-detailed in the work of (Huang et al. 2018). The new challenge in the corrosion inhibition arena today is that these natural substances must replace the synthesized organic inhibitors. Therefore, the present study investigates the possibility of substituting this agro-waste inhibitor with chemical inorganic and organic formulations to promote environmental sustainability. The results from this study indicated that PKSE is a promising candidate for inhibiting the corrosion of carbon steel in acidic media.

## Experimental procedure

### Materials and extract preparation

The test samples were prepared from 1018 low carbon steel rod with the following elemental compositions (wt%): Si (0. 35), S (0.035), C (0.18), P (0.03), Mn (0.77), and Fe (98.6) that were readily available in the market. The samples were prepared according to ASTM Standards (ASTM G1-03 2011), and NACE International Standards (2000). The carbon steel specimens were metallographically characterized by polishing with silicon carbide paper and fine polishing with alumina suspensions (0.05 μm). The coupon surface was polished mechanically: abraded with emery paper up to 1200 grade, washed and degreased with distilled water and ethanol, and finally dried until the surface exhibited mirror-like reflection. Thirty-seven percent hydrochloric acid purchased from Merck company was utilized to prepare the 1.0 M HCl solutions by diluting in double distilled water and used as the corrosion solution (Abdallah et al. 2023).

#### Extraction of inhibitor

The dried palm kernel shell was collected from Gauteng, South Africa. The shell was washed, filtered thoroughly to remove sand and other particles, air-dried, and then powdered with a grinder (Sanni et al. 2022a). The palm kernel shell powder was kept in the container protected from light before the corrosion test. Two hundred fifty milliliters of 98% ethanol was added to 100 g of palm kernel shell powder in a glass flask and afterward refluxed for 48 h (Kaur et al. 2024). The extracts were filtered, and a rotary evaporator was used to remove the excess ethanol. The PKSE were soluble in aqueous acid medium. The toxicity assessment of palm kernel shell was detailed in the work of (Mahmud et al. 2020). For the corrosion tests, the 1 M HCl without PKSE, referred to as the blank solution, and various concentrations ranging from 100 to 500 ppm were prepared as test media.

### Corrosion tests

#### Weight loss measurement

The electrochemical and weight loss test was conducted on the 1018 low carbon steel surface. The 1018 steel was cut into samples with (5 × 1.2 × 0.2) cm dimensions. The weight loss measurements were conducted based on (NACE TM0169/G31). A small hole was created at the center of the weight loss samples to pass a thread through it. The specimen utilized for the electrochemical test was Cu-wire soldered and sealed with epoxy resin for electrical connections with a 1.0 cm^2^ surface area. The sample surface was purified with different emery papers (400, 600, 800, 1000, and 1200) before putting the samples into the solutions to get a smooth surface. Afterward, the sample was washed to eliminate contaminants from the previous steps. The sample was then suspended in the corrosive solutions in the open air. After each incubation time, the corrosion rust formed on the metal surface was gently removed with a bristle brush. Afterward, the sample was washed, dried, and reweighed. The CR (corrosion rate) was estimated according to Eq. 1 (Sanni et al. 2023):

1 $$\text{CR}(\text{mm}/\text{year})=\frac{87.6\text{WL}}{\text{DAT}}$$

where WL is the weight loss (mg), *D* is the density (g/cm^3^), and *T* is the exposure time (hours). The difference between the initial and final weight was used to estimate the weight loss. IE (percentage inhibition efficiency) of PKSE was estimated using Eq. 2:

2 $$\text{IE}=\frac{{\text{CR}}_{(\text{o})}-{\text{CR}}_{(\text{Inhibitor})}}{{\text{CR}}_{(\text{o})}}\times 100$$

where CR_(Inhibitor)_ and CR_(o)_ are the corrosion rate value of the carbon steel in 1 M HCl with and without PKSE, respectively.

#### Electrochemical measurement

The electrochemical test was conducted in three conventional electrode cells with carbon steel samples as the working electrode, saturated Ag/AgCl as the reference electrodes, and platinum rods as counter electrodes. The electrochemical technique was conducted according to ASTM G1-03. The potential was scanned in the range of ± 250 mV relative to corrosion potentials at a scan rate of 1 mV/s (Sanni et al. 2023) in the potentiodynamic polarization test. EIS curve was recorded at the OCP with 10 kHz to 0.01 Hz frequency ranges via Frequency Response Analysis software. The Tafel extrapolation test was utilized to obtain the relative parameters. The linear Tafel segment of the cathodic and anodic curve was extrapolated to the *E*_corr_ (potential) to obtain the *i*_corr_ (current densities). To evaluate the reproducibility, all experiments were repeated three times at each concentration.

### Evaluation of tribological properties

1018 low-carbon steel was the material utilized in this experiment. The normal load was applied to the device by hanging weight on the bearing levers where the carbon steel samples are attached to produce the loads desired. A low engine-speed interval was chosen during the test because such a condition generates the highest friction, especially during the first movement and at the top dead center (Sakinah et al. 2016). The operating time was 10 min per sample at room temperature. The coefficient of friction was estimated using Eq. 3 (Sakinah et al. 2016).

3 $$\mu k={~}^{Fk}\!\left/ \!{~}_{N}\right.$$

where μ*k* is the kinetic friction coefficient, *Fk* is applied force, and *N* is the load. Equation 4 was used to evaluate the specific wear rate:

4 $$\Delta w=(w1-w2)$$

where Δ*w* is the specimen weight loss, *w*1 is the specimen weight before the test, and *w*2 is the sample weight after the test. The sample volume loss (Δ*V*) is estimated with Eq. 5:

5 $$\Delta V=\frac{1}{\rho }(w1-w2)$$

where ρ is the sample density. The samples specific wear rate (*ws*) was computed according to Eq. 6:

6 $$ws=\frac{\Delta v}{FnXSs}$$

where *Fn* is normal load (N) and S*s* is the sliding distance (m).

### Adsorption isotherm

Additional information on the substance properties under investigation can be found with the adsorption isotherms. Several adsorption isotherms, including Langmuir (7), Temkin (8), and Frumkin (9), should be utilized to estimate the inhibitor’s degree of surface covering (*θ*) in order to choose the best isotherm model. Hence, the degree of surface coverage for various inhibitor at different concentrations in 1 M HCl solution was examined using weight loss techniques.

7 $$\frac{C}{\theta }=\frac{1}{{K}_{\text{ads}}}+C$$

8 $$\text{log}\frac{\theta }{C}=\text{log}K+\alpha \theta$$

9 $$\text{log}\frac{\theta }{1-\theta }=2.303\text{log}K+2\alpha \theta$$

where *K*_ads_ is the adsorption–desorption constant and *θ* is the surface coverage.

### Surface morphology analysis

#### FTIR analysis

FTIR is a non-invasive, applicable, and relatively simple method for studying the influence of extracts on inhibitory characteristics (Hembram et al. 2018). The presence of different elements on the carbon steel surface was observed by FTIR analysis. The analysis was performed by Fourier Transformed Infrared Spectroscopy made by PerkinElmer in the range of 500–4000 cm^−1^. This technique relies on the fact that infrared light absorbed by a sample will be converted into heat. The absorption and transmittance values of the sample might be used to determine its identity from the spectral data.

#### SEM and EDX analysis

The scanning electron microscope may be used to gather information on the nanoscale surface morphology of particles. The corrosion morphology and compositions of the corrosion product were studied by scanning electron microscope (JSM 7800F, JEOL, Oxford) (SEM) and energy dispersive X-ray spectroscopy (EDX) for the carbon steel elemental mapping. The tests were performed at the accelerating voltage of 20 kV. The importance of EDX in extract characterization for identifying the elements present in a sample has been well established in the literature (Chowdhury et al. 2023). An energy dispersion x-ray spectroscopy is performed for the same sample to get a relative quantitative measure of elemental concentration. Hence, in this work, the inhibitory effect of PKSE was evaluated using the potentiodynamic polarization, weight-loss, and EIS techniques.

## Results and discussion

### Weight loss test

**Fig. 1:**
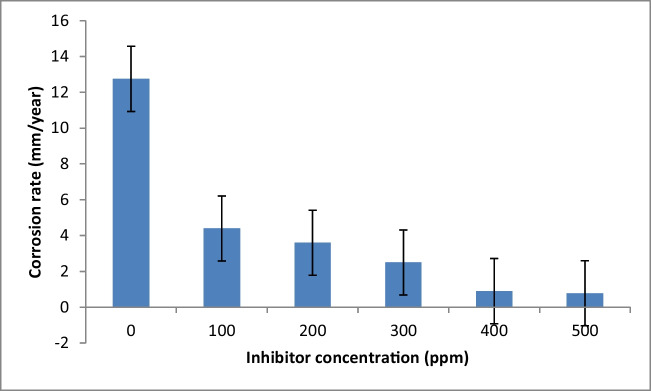
Corrosion rate for carbon steel in 1 M HCl solution with and without different concentrations of PKSE at 298 K

**Fig. 2:**
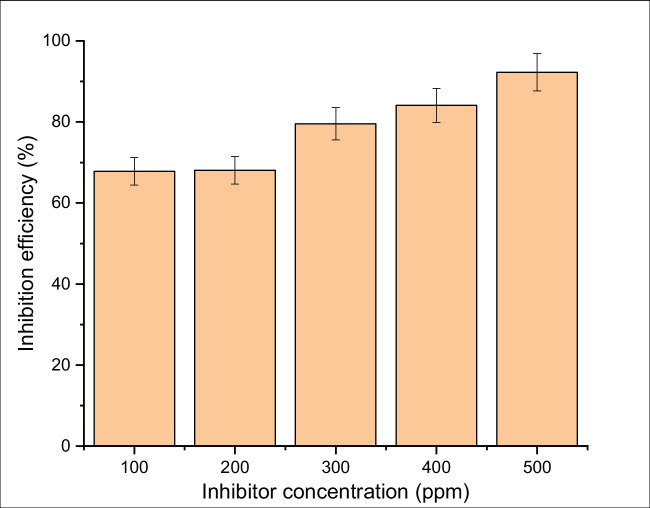
Inhibition efficiency for carbon steel in 1 M HCl solution with and without different concentrations of PKSE at 298 K

The weight-loss technique is considered a credible and accurate means to assess the corrosion rate of metals. The test was conducted for the PKSE at 298 K in the 1 M HCl medium. The specimens were hung with thread and dipped in beakers with 250 ml of 1 M HCl in the absence and presence of PKSE. Figures 1 and 2 illustrate the relationship between PKSE concentration and corrosion rate with the corresponding inhibition efficiency at various concentrations. Inspecting Fig. 1 and 2 indicates that the PKSE acts as an efficient inhibitor for the carbon steel at all concentration range studied (100–500 ppm). A small amount of PKSE addition to the corrosive solution significantly enhanced the corrosion resistance. At high PKSE concentration, the corrosion rate was significantly decreased (Fig. 1). The inhibitor efficiency increases with an increase in the PKSE concentration (Fig. 2). The increase in inhibition efficiency with inhibitor concentration indicates that more inhibitor molecule is adsorbed on the surface of the carbon steel at higher concentration, thereby leading to higher surface coverage. This observation is similar to the ones reported by Jafari et al. (2022), Wang et al. (2022a), and Beniken et al. (2022). The corrosion rate of the inhibited sample decreases by approximately 79.56%, and with the increase in concentrations, the corrosion rate decreases at a faster rate. The highest inhibition efficiency was obtained at 500 ppm concentrations of the PKSE as shown in Fig. 2. This result revealed that PKSE can be utilized as a sustainable inhibitor to increase the corrosion resistance property of the metal in hydrochloric acid environments (Lgaz et al. 2020). The inhibition efficiency of PKSE shows this trend: the PKSE efficiency increases linearly with concentrations from 100 to 500 ppm but tends to stabilize above 200 ppm. At 300 ppm dosage of PKSE, the efficiency exceeds 79%. The inhibitive performance of PKSE was ascribed to the adsorption of this compound onto the surface of the metal. The inhibitor’s high potential could be clarified by the presence of lone pair electrons of O, S, and N atoms and π-rich systems, which interact strongly with the surface of the metal (Ikeuba et al. 2022). By increasing the PKSE concentration, the carbon steel corrosion rate is decreased, leading to increased percentage inhibition efficiency (IE). This observation is owing to the PKSE chemical components adsorbed on the metal surface (Galai et al. 2020). The PKSE adsorbed on the surface of the metal could be via interaction between the unoccupied orbital of carbon steel and the lone pair electrons. The as-formed film behaves as the prominent barrier for inhibiting the corrosion of carbon steel in HCl solution. After immersion in HCl aqueous solution for 120 h, the steel surface still shows high corrosion prohibition effect with high inhibition efficiency, hence, inhibiting the metal surface from corroding. The active inhibitor surface that is available to interact with the metal surface can be considered as a parameter affecting inhibition performance. Therefore, it could be expected that inhibitor with higher active surface area exhibits better inhibition efficiency. Similar results have been reported elsewhere (Jafari et al. 2022; Wang et al. 2022b; Beniken et al 2022; Lgaz et al. 2020; Ikeuba et al. 2022). This result could be ascribed to increased PKSE concentration in the more concentrated solution, which readily reacts with the Fe ions present in the solution. Zaher et al. (2022) investigated the effect of *Ammi visnaga* L. extract on carbon steel corrosion in one molar hydrochloric solution: the reduction in corrosion rate was significant (84%) with increased concentration at 700 ppm and a similar trend is reported in this study.

### Potentiodynamic polarization measurements

**Fig. 3:**
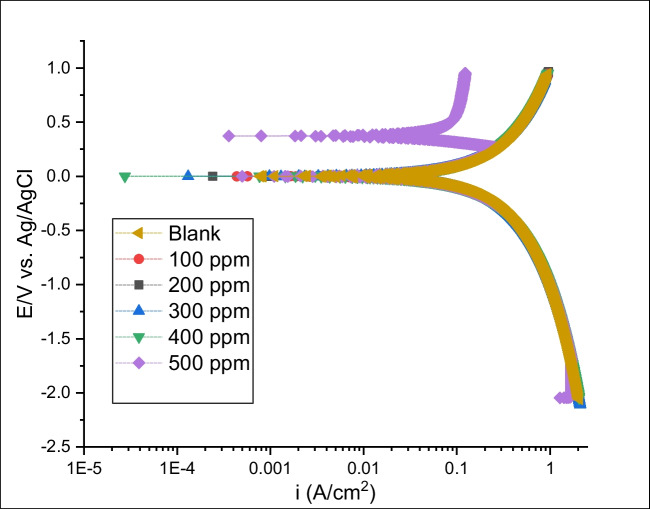
Polarization curves for carbon steel in hydrochloric acid solution

Potentiodynamic polarization (PDP) is an essential tool to understand corrosion inhibitor’s role. Figure 3 shows the PDP curves of carbon steel in hydrochloric acid solutions in the presence and absence of various PKSE concentrations. The corresponding plots of corrosion current density and corrosion potential were estimated from the polarization curve by Tafel extrapolations. To calculate the corrosion current density (*i*_corr_), polarization resistance (*R*_p_), and corrosion rate (CR), we use Tafel extrapolation method to analyze the data in Fig. 3 (Jiang et al. 2021). The corrosion current density (*i*_corr_) in Fig. 3 is calculated by superimposing the straight line along the linear portion of the cathodic or anodic curve and extrapolating it through *E*_corr_. The polarization resistance *R*_p_ is connected with the corrosion rate by Stern-Geary equation:

10 $$Rp=\frac{1}{2.303icorr\left(\frac{1}{ba+bc}\right)}$$

where *b*_*a*_ and *b*_*c*_ are the slopes of the straight lines along linear portion of anodic and cathodic curves, respectively.

The corrosion rate (CR) can be estimated by Eq. 11:

11 $$CR=\frac{0.13xicorrxE.\text{W}}{d}$$

where E.W is the equivalent weight of the oxidized elements and *d* is the density of the corroded specimen.

The polarization data at various concentrations obtained from Tafel extrapolation, i.e., cathodic and anodic Tafel slopes (*β*c, *β*a), corrosion potential (*E*_corr_), and corrosion current density (*i*_corr_), are reported in Table 1. The inhibited samples showed lower cathodic and anodic current density compared to the uninhibited sample, signifying better corrosion resistance (Damej et al. 2022). The corrosion current density value was estimated from this curve via Tafel extrapolation to quantify the corrosion resistance. From the PDP curve, it can be seen that the PKSE caused a decrease in the cathodic and anodic current density, most likely because of the adsorbed organic compound present in the PKSE at the electrode surface active sites. The corrosion rate estimated from the corrosion current densities for the inhibited sample at 100 ppm was estimated as 0.0007 mm/year, while at 500 ppm for the inhibited sample is 0.0003 mm/year. In contrast, the uninhibited sample exhibited a higher corrosion rate of 0.0056 mm/year. Also, the addition of PKSE shifted the curve to higher positive potentials, showing that samples with inhibitors are more corrosion resistant than the uninhibited samples. Consequently, it can be said that the anodic passivity is enhanced and stable anodic passive films are possibly formed on the carbon steel surface with PKSE (Kazantseva et al. 2022). As presented in Fig. 3, the PDP curve moves in the negative potential direction with smaller current densities, and it is worth noting that corrosion current densities reduced remarkably with the increase of PKSE compared with the uninhibited sample, meaning that corrosion gradually decreases with inhibitor. The result suggests that the inhibitor molecule is adsorbed in the solution/metal interface hindering the corrodent active sites. However, it is reported that the corrosion rate of carbon steel in the presence of 100–500 ppm of the PKSE is decreased with increasing the concentration of the green corrosion inhibitor utilized in this study. The more positive potential and lowest current density with the addition of PKSE offer the optimum corrosion resistance, hence, indicating the PKSE’s ability to retard the corrosion reaction. According to Raisemche et al. (2024), (i) if the *E*_corr_ displacement is < 85, the inhibitor is mixed-type, and (ii) if *E*_corr_ displacement is > 85 mV to blank, the inhibitor is regarded as anodic or cathodic type. In this study, the highest displacement is less than 85, indicating that PKSE is mixed-type. This result shows that the PKSE can retard both cathodic and anodic reactions. Tafel slope constants (*β*c and *β*a) do not significantly change in the inhibited system compared to the system without an inhibitor, that is, they can retard the cathodic hydrogen evolution reaction and reduce anodic Fe dissolution (Zhang et al. 2022).

**Table 1:** Electrochemical parameter for carbon steel in hydrochloric acid solution

Concentration (ppm)	*E*_corr_ (V)	*i*_corr_ (Acm^−2^)	*βa* (V/dec)	*βc* (V/dec)	CR (mm/year)
Blank	− 0.505	6.04E − 07	0.08	0.090	0.0056
100	− 0.511	4.82E − 07	0.019	0.023	0.0007
200	− 0.516	4.75E − 08	0.023	0.017	0.0006
300	− 0.522	3.48E − 08	0.037	0.040	0.0004
400	− 0.570	3.31E − 08	0.026	0.029	0.0004
500	− 0.600	2.73E − 08	0.026	0.039	0.0003

### Electrochemical impedance spectroscopy (EIS)

**Fig. 4:**
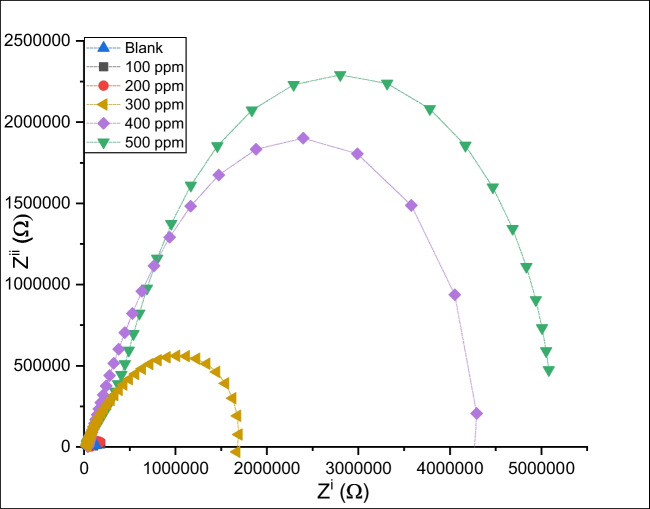
Nyquist plots for carbon steel corrosion in hydrochloric acid solution in the absence and presence of PKSE at 298 K

**Fig. 5:**
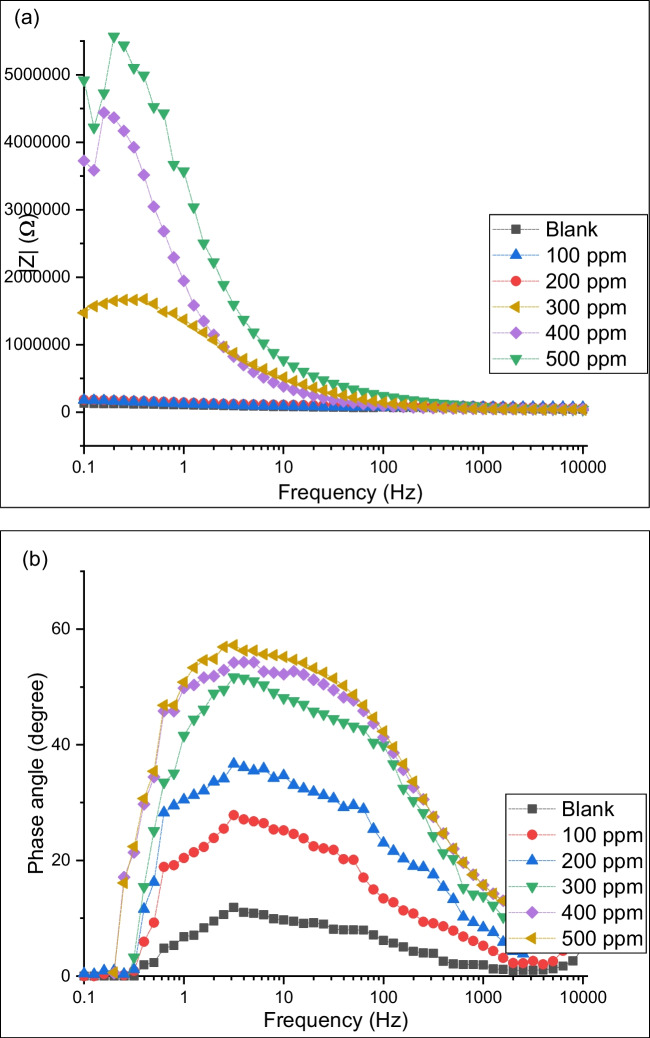
Effect of PKSE concentrations on the electrochemical behavior of carbon steel. **a** Bode plots; **b** impedance modulus plots

The EIS is an important and useful technology providing information about the electrochemical process kinetics that occurs at the metal surface interface and the corrosive solution to determine the corrosion rate. Figures 4 and 5 show the EIS plot and fitted curves for carbon steel in hydrochloric acid solutions at different PKSE concentrations.

**Fig. 6:**
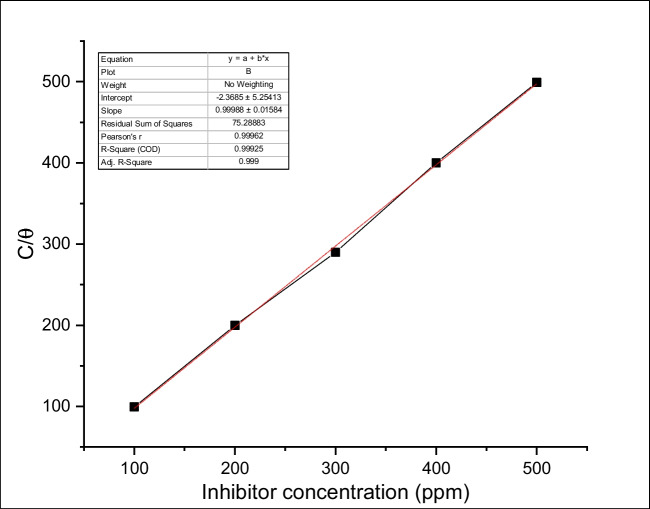
Langmuir isotherm plot for the corrosion inhibition mechanism on carbon steel surface with different concentrations of PKSE

Figure 6 shows the equivalent circuit for the obtained Nyquist plots. The applied constant phase element (CPE) instead of pure capacitors contains two specific parameters: *n* (the CPE exponent value between 0 and 1), and *Y* (CPE constant). The total *R* (oxide layer resistance and sum of the charge transfer resistance) and the CPE impedance are estimated based on the equations reported in (Vivier and Orazem 2022). The fitting result was in good agreement with the experimental data with less than 1% average error. Figure 4 shows a depressed Nyquist spectra semicircle, and the deviation from typical semicircles was ascribed to roughness and inhomogeneity of the metal surface (Zheng et al. 2022). The semicircle plots shown in Fig. 4 for inhibited carbon steel in the acid solutions are higher than the blank sample in the acid solutions; the semicircle diameter increases with PKSE concentrations. Generally, a bigger semicircle denotes higher inhibitive performance (Wang et al. 2022a). Hence, the result above indicates that the PKSE studied can lessen carbon steel corrosion (Tan et al. 2023). At high frequencies between 100 and 1000 Hz, the influence of fast electron-transfer results in a lower total impedance and higher phase for the highest inhibitor concentration as expected for higher electrolyte conductivity. At frequencies between 1 and 0.01 Hz, the total impedance and phase shift can be attributed to electrochemical processes near the metal surface (e.g., oxide layers) (Tan et al. 2024b).

As shown in Fig. 5b, the phase angle value is higher for the inhibited carbon steel in the hydrochloric solutions, with a wide frequency range, compared with the uninhibited sample. The high-frequency time constant could be attributed to capacitance and the resistance of the oxide film on the metal surface, and the low-frequency time constant is ascribed to the Faradic charge transfer process at the inhibitor/electrolyte interface. The presence of PKSE enlarges the semicircle diameter of the Nyquist plot without altering the plot shape. With PKSE, the semi-circle size increases progressively; this is normally associated with the charge transfer mechanisms, with an increased resistivity of the metal surface. Increasing the PKSE concentrations increases the charge transfer resistance drastically and the CPE strongly decreases. This observation could be described by the decrease in the local dielectric constant and/or the increase in the electrical double layer thickness (owing to the surface sorption of PKSE). This indicates that the inhibitive performance of PKSE is due to the adsorbed PKSE molecule on the carbon steel surface without the variation in the corrosion mechanisms. Such high potential of the PKSE could be explained by the presence of lone pair electrons of O atoms and π-rich systems that interact strongly with the metal surface.

The impedance spectra in the Bode diagram (Bode diagram is a useful way to represent the gain and phase of a system as a function of frequency) show low corrosion resistance of the carbon steel in the blank hydrochloric acid solutions (Fig. 5a). The resistance of the medium with PKSE decreases significantly. The highest phase angle in the solutions with PKSE at high frequency (Fig. 5b) is shifted to the right in contrast to its behavior in the reference corrosive solution. The *n* value with PKSE was higher than the uninhibited sample, and this value increased from 0.77 to 0.91 with an increase in the PKSE concentrations from 100 to 500 ppm. It was observed that the PKSE increases the charge-transfer resistance *Rct* of sample in hydrochloric acid solutions by a factor of 2.6, with the use of the EIS Spectrum Analyzer computer program. The widening of the phase angles (Fig. 5b) at the intermediate frequency by the addition of PKSE confirms the PKSE molecules’ film adsorption on the carbon steel surface. Similar behavior was observed for corrosion inhibition of berberine (Li 2019), curcuma (Florez-Frias et al. 2021), and coconut shell extracts (Sanni et al. 2022b). The Rct values increase with the PKSE concentration. This signifies that the impedance of carbon steel surface to the flow of charges across the metal and electrolyte interface increases with PKSE. Also, capacitance double-layer (Cdl) reduced value by addition of PKSE molecule is owing to the increase in the thickness of the electric double layer and/or decrease in the dielectric constant.

### Adsorption isotherm

The adsorption of inhibitor molecules onto the metallic surface, with water molecules displacement, can be explained by the inhibition mechanism for organic inhibitors in an aqueous solution, especially in acid solutions. The adsorption isotherm can be used to describe the interactive nature between the carbon steel surface and the PKSE molecule throughout the inhibition process. It is informative to investigate the possible mode of adsorption by testing the obtained experimental data with different adsorption isotherms in the situation where it is suspected that the metal corrosion inhibition occurred due to the inhibitor molecules’ adsorption onto the metal surface. The inhibitor efficiency depends on concentration and increases with PKSE concentrations; this is because more PKSE molecules would be adsorbed onto the surface of the metal at high PKSE concentration, thus enhancing inhibition efficiency and increasing surface coverage (*θ*) (Sanni et al. 2022a). Adsorption isotherms provide important information about the interactions and related thermodynamic values. In order to determine the best isotherm, it is assumed that the corrosion inhibition efficiency is linear relationship with the surface coverage (*θ*), and this values were derived from percentage inhibition efficiency values (Kaya et al. 2023).

In this study, obtained data from weight loss techniques was graphically tested for fitting different adsorption isotherms such as Freundlich (0.85), Frumkin (0.756), Langmuir (0.99925), and Temkin (0.7321). The Langmuir adsorption isotherms show the best fit for PKSE. This hypothesis is confirmed by the linear regression coefficient, which would be considerably large (0.99925) and near to 1 (Kaya et al. 2023). It is well known that the PKSE molecule has antioxidative property; this denotes that the PKSE molecule in the hydrochloric acid solution retards the hydrochloric oxidation and decreases the corrosive rate. Figure 6 shows the plot of (*C*inhibitor/*θ*) versus *C*inhibitor, and a linear relationship is obtained. The *K*ads were calculated from the intercepts of the straight lines. The regression coefficient *R*^2^ value close to one confirmed that the adsorption of PKSE on the carbon steel surface fits the Langmuir adsorption isotherms. The straight line slope is very close to one, denoting that the inhibitor molecule adsorbed forms monolayer on the metal surface (Sanni et al. 2018). The high adsorption equilibrium constant *K*ads value reflects the high inhibitor adsorption ability on the metal surface.

### Mechanical properties

**Fig. 7:**
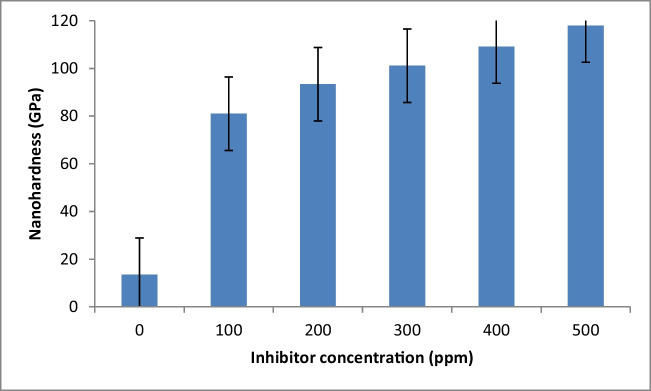
Nanohardness profiles for the carbon steel as a function of inhibitor concentration

**Fig. 8:**
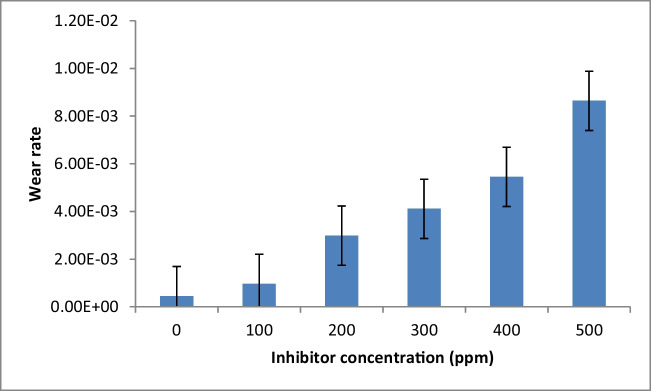
Wear rate variation as a function of inhibitor concentration

The elastic modulus and hardness of the inhibited samples were characterized by nanoindentation tests, and the obtained results are shown in Fig. 7. The increase of PKSE concentration from 100 to 500 ppm leads to a decrease in tensile stress value. The reduced tensile stress with PKSE arises probably because of the excess atoms in the PKSE film. The decreased tensile stress with PKSE film can be ascribed to the organic compounds forming composite PKSE film and incorporation of metal–oxygen. The mechanical property of the PKSE film obtained from nanoindentation tests is presented in Fig. 8. The material wear behavior is normally characterized by a long elastic strain to failure, as described in Fig. 8, indicating that the PKSE in this experiment possesses excellent mechanical properties. With increased PKSE concentration, the hardness shows an obvious decline trend. With 100 ppm PKSE, the hardness was 80.99 GPa. However, when the PKSE concentration increased to 500 ppm, the hardness of the inhibited sample increased to 118 GPa, which is related mainly to the inhibitor transformation from amorphous structures to crystalline structures. Leyland and Matthews (2000) believed that the high *H*/*E* ratio (that is hardness (*H*) is the main mechanical property which determines the wear resistance of materials. Young’s modulus (*E*) has also been shown to influence the wear resistance. The *H*/*E* ratio is often used as an index to represent wear resistances of materials and coatings) is normally a reliable indicator of good wear resistance. The calculated *H*/*E* value was found to increase with inhibitor concentration. The amorphous phase formed eliminates defects and micro-stress in the inhibitor and improves the inhibitor strength. The transition from amorphous states to crystalline states increases the number of grain boundaries in the inhibitor, which intensifies the blocking effects on the internal defect of the inhibitor and enhances the inhibited sample hardness (Lian et al. 2022).

### Surface analyses

**Fig. 9:**
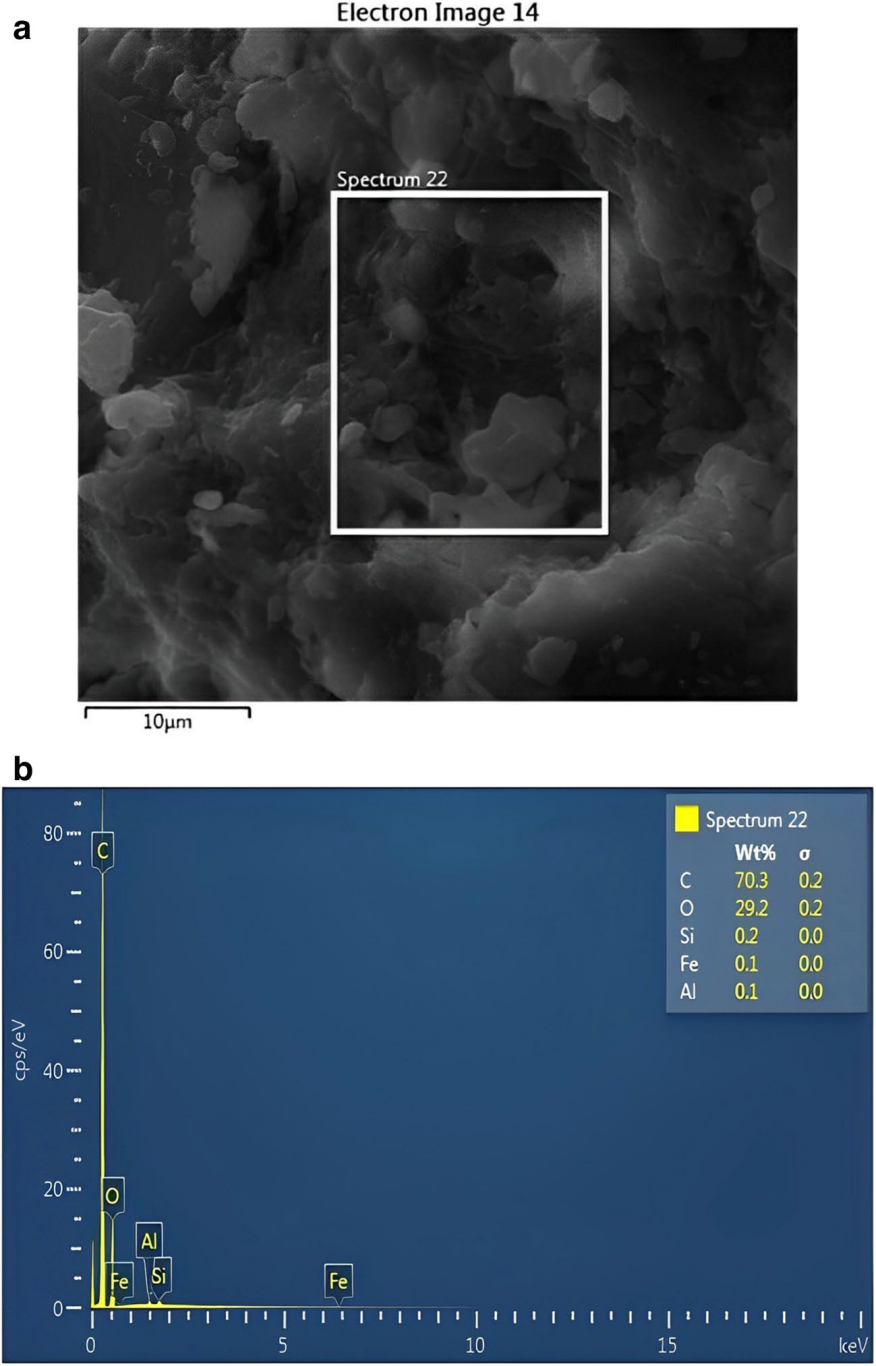
SEM/EDX of palm kernel shell extract

**Fig. 10:**
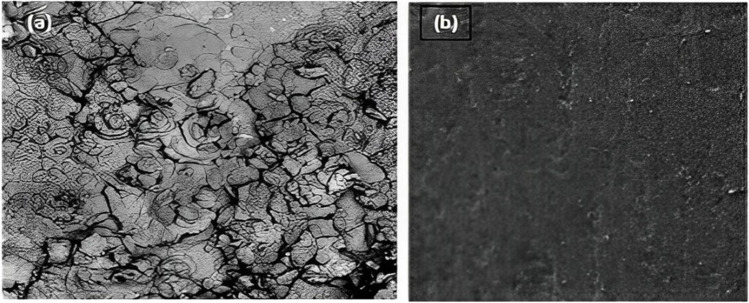
SEM image of the carbon steel after weight loss tests **a** without inhibitor and **b** with inhibitor

**Fig. 11:**
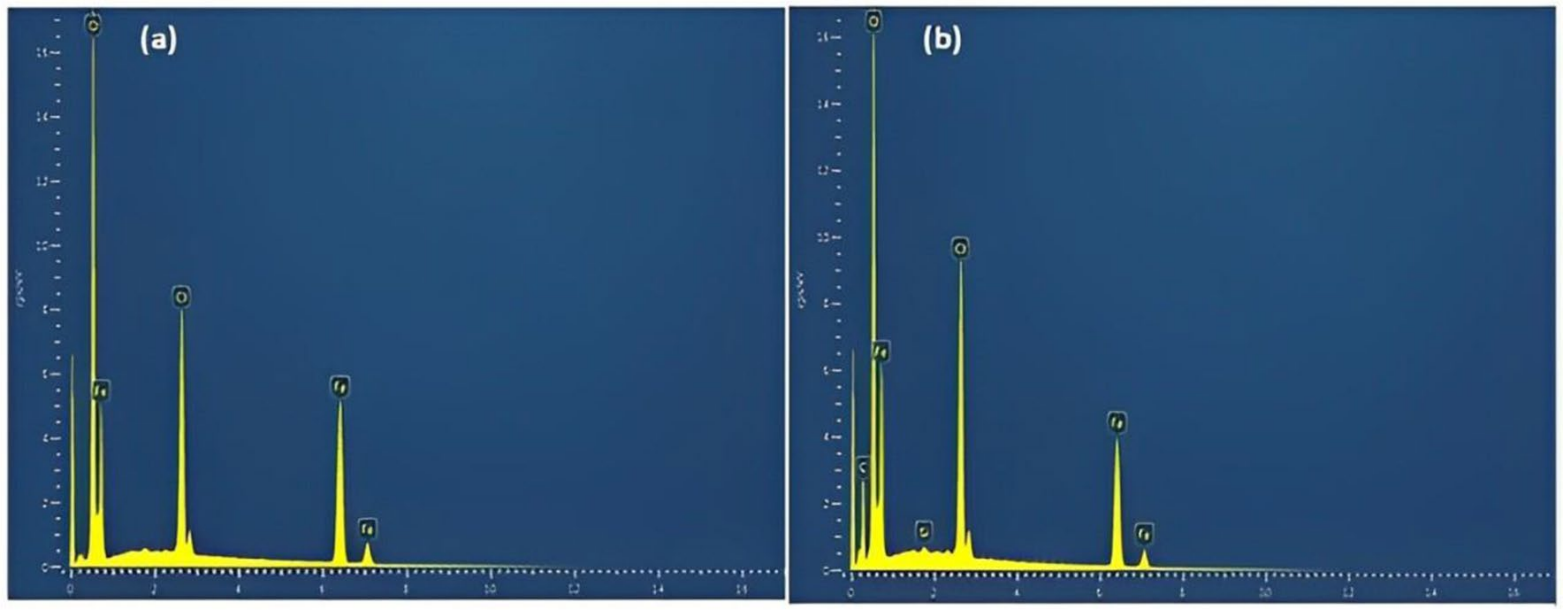
EDX analysis after weight loss test **a** without and **b** with inhibitor

The SEM and EDX of the PKSE used in this study are presented in Fig. 9. The SEM images in Fig. 10 visually compare the corrosion morphology with and without corrosion inhibitor. Figure 10a, b corresponds to the corrosion morphology without and with 500 ppm PKSE. Comparing Fig. 10 a and b, it was observed that the uninhibited steel surface becomes uneven and severely corroded in the hydrochloric solutions with deep cracks. Also, surface degradation owing to exposure to the acid solution can be seen, while there is less damage on the samples’ surface with 500 ppm PKSE. As depicted in Fig. 10b, this result shows that PKSE addition decreases the corrosion rate and the PKSE inhibition is effective. Figure 11 shows the elemental composition present on the electrode surface. An increase in the percentage of oxygen on the carbon steel surface is the reason for the formed corrosion products on the carbon steel surface. Decreased O and increased Fe in the1 M HCl solution with 500 ppm PKSE is the reason for the protective carbon steel substrate. Herein, SEM figures can confirm the electrochemical and gravimetric results and prove that the PKSE adsorption films play a crucial role in retarding the carbon steel corrosion in the hydrochloric acid solutions.

### Corrosion inhibition mechanisms

The possible inhibition behavior of PKSE was estimated using experimental methods and surface examination. The phytochemical analysis of the palm kernel was conducted by Ofori-Boateng and Lee (2013) and Tsouko et al. (2019); Ogbiede et al. (2022) also reported the same. Organic compounds in PKSE contain N and O atoms in their functional groups, such as C-O, O–H, C = O, N–H, and O-heterocyclic ring in their molecular structure. This property satisfies the property of a corrosion inhibitor. Therefore, this compound is responsible for the observed efficiency of PKSE on the carbon steel surface in the inhibitor-hydrochloric acid solutions. Carbon steel corrosion in an aqueous solution depends on the anion concentration in the solution. The corrosion mechanism of the carbon steel must be known, to predict the adsorption type.

The results obtained in this study indicate that PKSE exhibits effective corrosion inhibition for carbon steel and the inhibition performance was enhanced with increased length of the alkyl tail. These mechanisms show that the anodic reaction kinetic is influenced by two intermediates: the oxide involving the adsorbed inhibitor molecule and the hydroxyl adsorbed. The adsorption of palm kernel shell on the carbon steel surface can occur directly via donor–acceptor interaction between the free pair electrons of the heterocyclic atom and π-electrons and the vacant d orbital of surface iron atoms. The PKSE may be adsorbed on the carbon steel surface as a natural molecule. Electron sharing occurs amid the nitrogen, oxygen atoms, and the surface of the metal, thus decreasing the carbon steel corrosion rate.

**Fig. 12:**
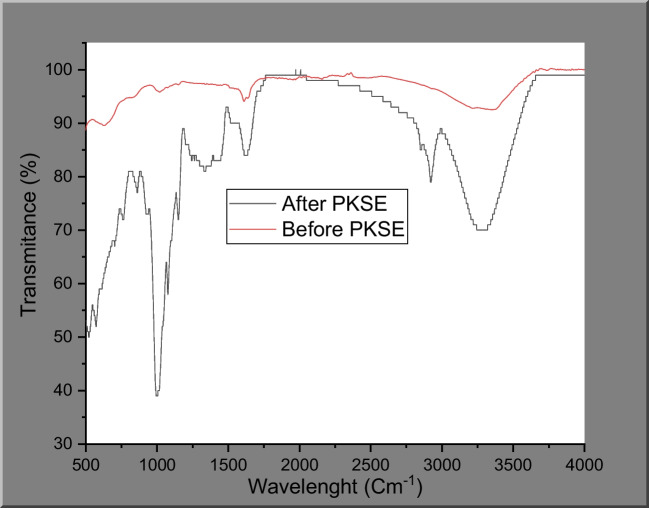
FTIR for PKSE in1 M HCl solution and for protective film formed on the carbon steel surface

The functional groups in the PKSE constituents were chemically identified via Fourier transform infrared spectroscopy (FTIR), as shown in Fig. 12. The broad peak centered at 3320 cm^−1^ is ascribed to the stretching vibration of O–H, resulting from the hydroxyl-terminated polyphenols and flavonoids (Abdelaziz et al. 2021). The peak at 2913 cm^−1^ is associated with the stretching vibration of the alkyl groups (–COOH) (Fan et al. 2020). The stretching of the typical C = C can be identified at 1637 cm^−1^. Another featured vibration for flavonoid is recognized at 1147 cm^−1^. The peak at 1019 cm^−1^ is the characteristic adsorption of the glycosidic structure (Solomon et al. 2018). According to the FTIR analysis, the oxygen heteroatoms, aromatic rings, and conjugated unsaturated bonds in PKSE can behave as active sites for adsorption on the C-steel surface and thus inhibit dissolution of the metal in the acidic media. FTIR spectrum of the protective film is also shown in Fig. 12 after immersion in 1 M HCl the in presence of 500 ppmml/PKSE at 60. With decreased transmittance, somewhat comparable spectrums were obtained, and shifts in transmittance were caused by interactions between the steel surface and the PKSE molecules.

### Comparative study of the PKSE extract

Corrosion inhibitors are considered as one of the most economical and effective means of protecting metals from corroding. Integrated waste valorization is a progressive form of resource conservation due to its environmental and economic values. In almost every country in the world, the extraction of organic compounds from waste has become a significant concern in waste management. Searching for cost-effective, efficient, and environmentally friendly corrosion inhibitors in aggressive media is a continuous process. In this study, we tried to propose PKSE as an inhibitor that satisfies the above-mentioned qualities. PKSE showed a high inhibition efficiency of 95.3% in 1 M HCl solution; to confirm the inhibition performance of PKSE, its efficiency was compared with the results of other authors. As can be seen in Table 2, the PKSE presented excellent inhibition efficiency on carbon steel corrosion in 1 M HCl solution.

**Table 2:** Comparing the efficiency of PKSE agro-industrial waste against other inhibitors in similar conditions

Compound	Average efficiency	Media	Concentration	Reference
Pistachio shell extract	92%	1 M HCL	800 ppm	Shahmoradi et al. 2020
Zea mays hairs waste extracts	87.92%	1 M HCl	0.15 g L^−1^	Aourabi et al. 2020
watermelon rind extract	46.12%	Artificial saliva	1000 ppm	Nahusona and Koriston 2019
Punica granatum extract	83%	0.05 M HCl	10 µM	Magni et al. 2020
Walnut fruit green husk extract	95%	1 M HCl	800 ppm	Shahmoradi et al. 2021
*Ircinia strobilina* crude extract	82%	1 mol L^−1^ HCl	2.0 g L^−1^	Fernandes et al. 2019
Spirogyra algae extract	93.03%	0.5 M HCl	2 g L^−1^	Verma and Khan 2016
Peach pomace extract	88%	0.5 M NaCl	800 ppm	Vorobyova and Skiba 2021
Purple onion extract	67%	0.5 M HCl	2.20 g L^−1^	Galo et al. 2021
Jujube shell extract	91%	1 M HCl	2 g/L^−1^	Jmiai et al. 2021
Tea waste extract	83.6%	1 M HCl	500 mg L^−1^	Pal and Das 2020
*Ammi visnaga* L. extract	84%	1.0 mol/L HCl	700 ppm	Zaher et al. 2022
Palm kernel shell	95.3%	500 ppm	95.3%	This work

## Conclusion

The use of palm kernel shell extract (PKSE) as a corrosion inhibitor for 1018 low carbon steel was assessed in 1 M HCl solutions using weight loss, electrochemical impedance spectroscopy, and polarization methods. The important conclusions derived from the study are summarized as follows:

- ✓ PKSE shows excellent inhibitive performance for carbon steel in 1 M HCl solutions, and an increase in its concentration gives a noticeable improvement in the inhibition efficiency.
- ✓ The weight-loss test demonstrates inhibition efficiency of 95.3% at 500 ppm concentration of PKSE. Electrochemical measurements indicated that PKSE in the acidic environment acts as a mixed-type inhibitor.
- ✓ The Langmuir adsorption isotherm represents the PKSE adsorption on the carbon steel surface, denoting that the PKSE molecule obscured a classic adsorption region when deposited on the metal/solution interface.
- ✓ The surface analysis via SEM technique confirmed the protective films formed by the PKSE component on the carbon steel.
- ✓ The FTIR analysis also confirmed the adsorption of PKSE on the carbon steel surface. According to SEM data, the adsorption of the inhibitor on the metal surface reduces surface damage. The outcome of this study confirmed the primary benefit of using agricultural waste-based corrosion inhibitors for protecting metal.

## Data Availability

The data will be made available on reasonable request.
